# The Dynamics of Disease Progression in Cystic Fibrosis

**DOI:** 10.1371/journal.pone.0156752

**Published:** 2016-06-01

**Authors:** Frederick R. Adler, Theodore G. Liou

**Affiliations:** 1 Department of Mathematics and Department of Biology, University of Utah, Salt Lake City, UT, United States of America; 2 Department of Internal Medicine, University of Utah, Salt Lake City, UT, United States of America; Laurentian, CANADA

## Abstract

In cystic fibrosis, statistical models have been more successful in predicting mortality than the time course of clinical status. We develop a system of partial differential equations that simultaneously track mortality and patient status, with all model parameters estimated from the extensive and carefully maintained database from the Cystic Fibrosis Foundation. Cystic fibrosis is an autosomal recessive disease that leads to loss of lung function, most commonly assessed using the Forced Expiratory Volume in 1 second (FEV1%). This loss results from inflammation secondary to chronic bacterial infections, particularly *Pseudomonas aeruginosa*, methicillin-sensitive *Staphylococcus aureus* (MSSA) and members of the virulent *Burkholderia* complex. The model tracks FEV1% and carriage of these three bacteria over the course of a patient’s life. Analysis of patient state changes from year to year reveals four feedback loops: a damaging positive feedback loop between *P. aeruginosa* carriage and lower FEV1%, negative feedback loops between *P. aeruginosa* and MSSA and between *P. aeruginosa* and *Burkholderia*, and a protective positive feedback loop between MSSA carriage and higher FEV1%. The partial differential equations built from this data analysis accurately capture the life-long progression of the disease, quantify the key role of high annual FEV1% variability in reducing survivorship, the relative unimportance of short-term bacterial interactions for long-term survival, and the potential benefits of eradicating the most harmful bacteria.

## Introduction

In some diseases, patient status can be usefully characterized by a single measurement that serves as a powerful predictor of mortality. Examples include prostate specific antigen (PSA) [1], MELD scores in liver transplantation [2], the Glomerular Filtration Rate in the kidney [3], and CD4 T cell counts in HIV [4]. Accurate mathematical models must capture a joint process of longitudinal change in this measurement and mortality [5], potentially coupled to transitions between discrete patient states, such as the acquisition or loss of a pathogen. Statistical methods for addressing this challenge have focused on identifying the key covariates linked to rapid decline or death, and correcting for patient differences as random effects [6–8].

We here develop a mathematical model for these joint dynamics (see related work on PSA with a Chapman-Kolmogorov equation [1, 9] or as a stochastic differential equation [10]). This approach provides two main benefits. First, model parameters can be estimated directly from existing longitudinal data, and the key relationships displayed graphically. Second, these models can be efficiently solved to study the population-level impacts of changes in parameter values, structural assumptions, and treatment.

Cystic fibrosis is an autosomal recessive disease characterized by a loss of lung function, typically measured by the Forced Expiratory Volume in 1 second (FEV1) as corrected to a percent of normal values, based on age, height, gender, race and ethnicity (FEV1%). Declines in FEV1% are due largely to the interaction between chronic bacterial infections and the inflammatory immune response evoked to combat them [11]. Although many microbes infect the CF lung [12], three bacteria have long been recognized as important. *Pseudomonas aeruginosa* is an opportunistic, highly prevalent gram-negative bacterium that associates strongly with acute exacerbations in CF patients [13], reduces the diversity of the CF lung microbiome [12, 14, 15], and is a primary target of treatment [16]. Methicillin-sensitive *Staphylococcus aureus* (MSSA) is an opportunistic, highly prevalent gram-positive bacterium with evidence of positive effects on survivorship [17], thought to be due in part to its strong negative association with *P. aeruginosa*[16, 18, 19]. The *Burkholderia* complex consists of a much less prevalent but often deadly set of gram-negative bacteria that infect few humans other than those with cystic fibrosis [20, 21].

Although FEV1% is a powerful predictor of mortality, predicting its own temporal dynamics has proven difficult [17]. Several models have addressed the problem of jointly modeling FEV1% change and survival, using statistical methods that indicate that rapid FEV1% decline has predictive power for survival not captured by FEV1% itself [22, 23]. Other studies have identified covariates associated with more rapid decline, including carriage of *P. aeruginosa*, MSSA and *Burkholderia* [8, 23–27], although the specific covariates identified differ among studies [28]. In turn, low FEV1% has been shown to predict an increased rate of acquisition of *P. aeruginosa* [29].

Our goals is to build a model of how patients change over their entire lives with parameters estimated from annual changes in patient status as recorded in the extensive and carefully maintained Cystic Fibrosis Foundation Patient Registry (CFFPR) [30, 31]. Because our data only record positive or negative cultures for the three bacterial infections, we refer to a change from a positive test in one year to a negative test in the next as “loss”, and a change from a negative test in one year to a positive test in the next as “acquisition,” although many of the observed changes do not describe permanent clearance or establishment of chronic infection.

We organize the presentation around two sets of predictions. The first set addresses the annual changes in patient status estimated from CFFPR data.

S1: Patients with lower FEV1% will have lower survivorship,
S2: Patients with lower FEV1% will be more likely to acquire *P. aeruginosa* and *Burkholderia*, and less likely to acquire MSSA,
S3: Patients with lower FEV1% will be less likely to lose *P. aeruginosa* and *Burkholderia* and more likely to lose MSSA,
S4: Patients with *P. aeruginosa* or *Burkholderia* will have higher mortality at any given FEV1% and a greater average annual reduction in FEV1%,
S5: All bacteria will have negative interactions, so that patients carrying any particular one will be less likely to acquire, and more likely to lose, either of the others.

The second set addresses the lifelong course of disease as predicted by the mathematical model (H1–H4).

H1: The model can accurately capture the dynamics of survivorship and FEV1% over the entire lifetime of patients without adjusting any free parameters,
H2: High variance in year-to-year values of FEV1% in individual patients is an important factor in reducing survivorship,
H3: Elimination of *P. aeruginosa* and *Burkholderia* will lead to significant improvement in survivorship and FEV1%, and elimination of MSSA will have the opposite effect,
H4: Removing the interaction between *P. aeruginosa* and MSSA will predict poorer outcomes by removing the protective effects of MSSA [17, 32].

Clarifying alternative hypotheses for the mechanism by which MSSA provides protection is one of our central goals. At one extreme, MSSA carriage could be merely a marker of better patient status. Alternatively, it could be protective solely through its antagonistic relationship with *P. aeruginosa*, thus preserving FEV1% and improving survival. At the other extreme, MSSA could have protective effects beyond those created by its antagonism with *P. aeruginosa*.

## Methods

Our data come from the CFFPR, which includes longitudinal information on patients from 117 certified cystic fibrosis centers in the United States during the period from 1986 through 2011 [30, 31]. Patients are evaluated for lung function (including FEV1), microbiology (including for *P. aeruginosa*, MSSA, and *Burkholderia*) and other clinical parameters during regular clinic visits and as needed during exacerbations, with a target of four measurements per year. FEV1 is difficult to measure in patients younger than 6 years of age, thus these patients are not included in this study. Microbiology is determined by bacterial culturing. Carriage of *P. aeruginosa*, MSSA, and *Burkholderia* is recorded based on at least one positive culture during a year. Prior to 2003, CF centers recorded an annualized result for each organism that indicated at least one positive culture during the year. Since 2003, multiple results are recorded, and we annualized these data in the same way to reflect if any culture in a year was positive. Measurements are not evenly spaced, but we treat each interval as being a single year, and include for analysis all pairs of consecutive years with data.

Due to the large changes in FEV1% associated with transplant, patients with a transplant in the current or subsequent year are excluded. Because survivorship models for patients differ greatly after transplant [33], we also exclude this set of patients from the current analysis. As this is an analysis of annual transitions, we do not introduce a bias by censoring patients at transplant, but may introduce biases by excluding some of the sickest patients from inclusion.

The main models use data from 1996–2011 to represent the current treatment era, with earlier data from 1986–1995 creating a comparison group. Raw FEV1 measurements were normalized to percent predicted FEV1 (FEV1%) [34]. We use the maximum value in each year as the most consistently reproducible value, which also matches the pattern in the early years of the CFFPR of reporting the best FEV1 in a year when reporting by centers was done on only an annual basis. Lower values, often measured during an acute exacerbation, vary widely depending on severity and stage of each exacerbation and are not easily reproduced [17]. To test for robustness, we repeated the analyses using the mean FEV1% in each year. Of the patient years included in our analysis, 31.8% have a single measurement, 14.9% have two measurements, 17.6% have three measurements, and 35.7% have four or more measurements.

The models are built from analysis of annual change in FEV1% (ΔFEV1%) and bacterial carriage, and the probability of death as a function of the current FEV1% and bacterial carriage. We split the population into eight groups based on carriage of *P. aeruginosa*, MSSA, and *Burkholderia*. We divide groups that exceed 10,000 data points into 100 equally-sized subgroups based on quantiles of FEV1%, and smaller groups into 10 subgroups, with numbers of patient-years indicated in Table 1).

**Table 1:** Number of patients in different bacterial carriage subgroups in the 1996–2011 CFFPR.

*P. aeruginosa*	MSSA	*Burkholderia*	Number of patient-years	Number of FEV1% subgroups
0	0	0	29331	100
1	0	0	76670	100
0	1	0	47085	100
1	1	0	54914	100
0	0	1	1855	10
1	0	1	2265	10
0	1	1	1769	10
1	1	1	1670	10

We use regression (the lm package in R [35]) of transformed outcomes to estimate the coefficients of the models (S1 Table, with those for patients before 1996 in S2 Table). The regressions use the mean FEV1% in the subgroup as the independent variable, and the mean outcome as the dependent variable. We use model selection to choose whether and how to transform data for these fits.

The outcomes fall into three categories: mortality, change in FEV1%, and change in bacterial carriage state. **Mortality:** We regress the observed mortality in each subgroup (deaths per year) against FEV1%, increased by the reciprocal of the median number of patients per subgroup to avoid zeroes. Log transformation of both variables provided the best fit. **Change in FEV1%:** We model the mean and variance of annual change in FEV1% with piecewise linear functions with a break point at FEV1% = 90% for each of the eight bacterial carriage states (slopes designated with subscripts *s* and *s*2 in S1 and S2 Tables). This break point was chosen as the approximate mean of break points found with piecewise linear regression (package segmented in R), matching the lower limit of normal for FEV1%. Because few patients with high FEV1% test positive for *Burkholderia*, we find a single slope with high FEV1% for the four groups with a positive test. **Bacterial acquisition and loss:** The probability of acquisition of each bacterial species, defined as the fraction changing from a negative to a positive test in one year, varied linearly in FEV1% without transformation for all three bacteria. The logarithm of the annual probability of loss of *P. aeruginosa* and *Burkholderia* increased linearly in FEV1%, and the untransformed annual probability of loss of MSSA decreased linearly in the logarithm of FEV1%.

We also derive several lower dimensional models for comparison. The first tracks only FEV1% without including any of the bacterial infections. A second set of three models tracks FEV1% in combination with each of the bacteria individually, breaking patients into two rather then eight bacterial carriage states. The third set examines the effects of bacterial interactions in two ways. A model with no bacterial interactions estimates the probability of acquisition of each bacterial species as a function only of FEV1%, without regard to the other two bacteria. Because of the known importance of the interaction between MSSA and *P. aeruginosa*, we also derive a model that excludes only this interaction [12]. In this case, we find the probabilities of acquisition and loss of MSSA and *P. aeruginosa* as functions of FEV1% and current carriage of *Burkholderia* only.

**Fig 1:**
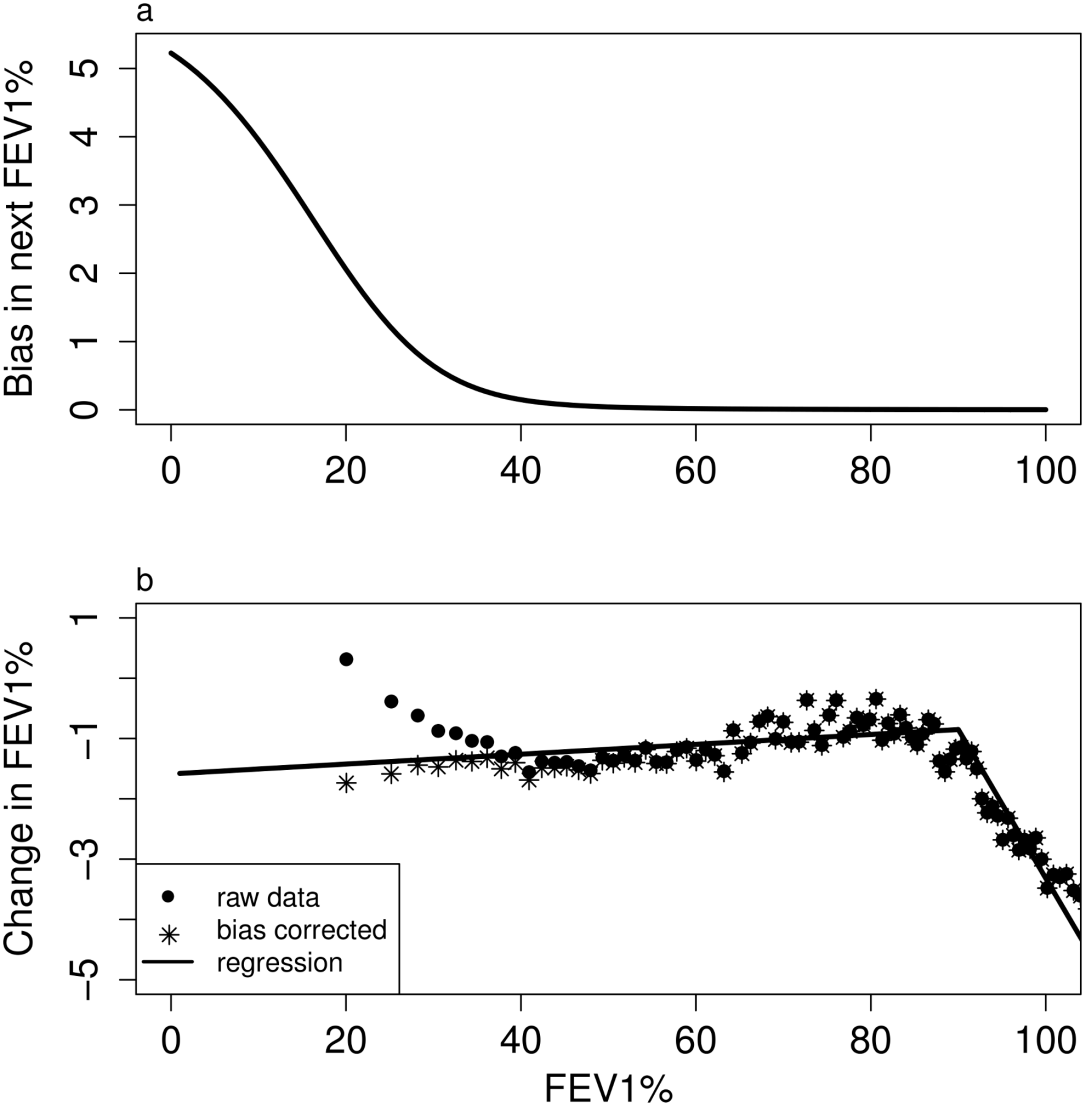
Correcting for survivorship bias. **a.** Excess estimated FEV1% in the following year due to mortality of patients with low current FEV1%. **b.** Change in FEV1% for all patients pooled estimated without (circles) and with (stars) correction for survivorship bias, compared with the regression fit.

The estimated change in FEV1% is altered by survivorship bias, with an increase in FEV1% observed in surviving patients with low FEV1% in the current year. We correct for this bias by creating a normally distributed population with the observed variance in ΔFEV1% as a function of current FEV1%. We pool over all bacterial carriage states because they have only a small effect on this variance. The bias is the increase in the mean of the next FEV1% created by the death of patients with low FEV1% (Fig 1a), which corrects for the deviation between the regression model and the data (Fig 1b).

These analyses of annual change in patient state as a function of current state provide the complete set of parameters for our mathematical model. To write as a system of partial differential equations, we transform to continuous time. An event (death, or acquisition or loss of one of the bacterial species) that occurs with probability *p*, corresponds with an annual rate *r* = −ln(1 − *p*).

**Fig 2:**
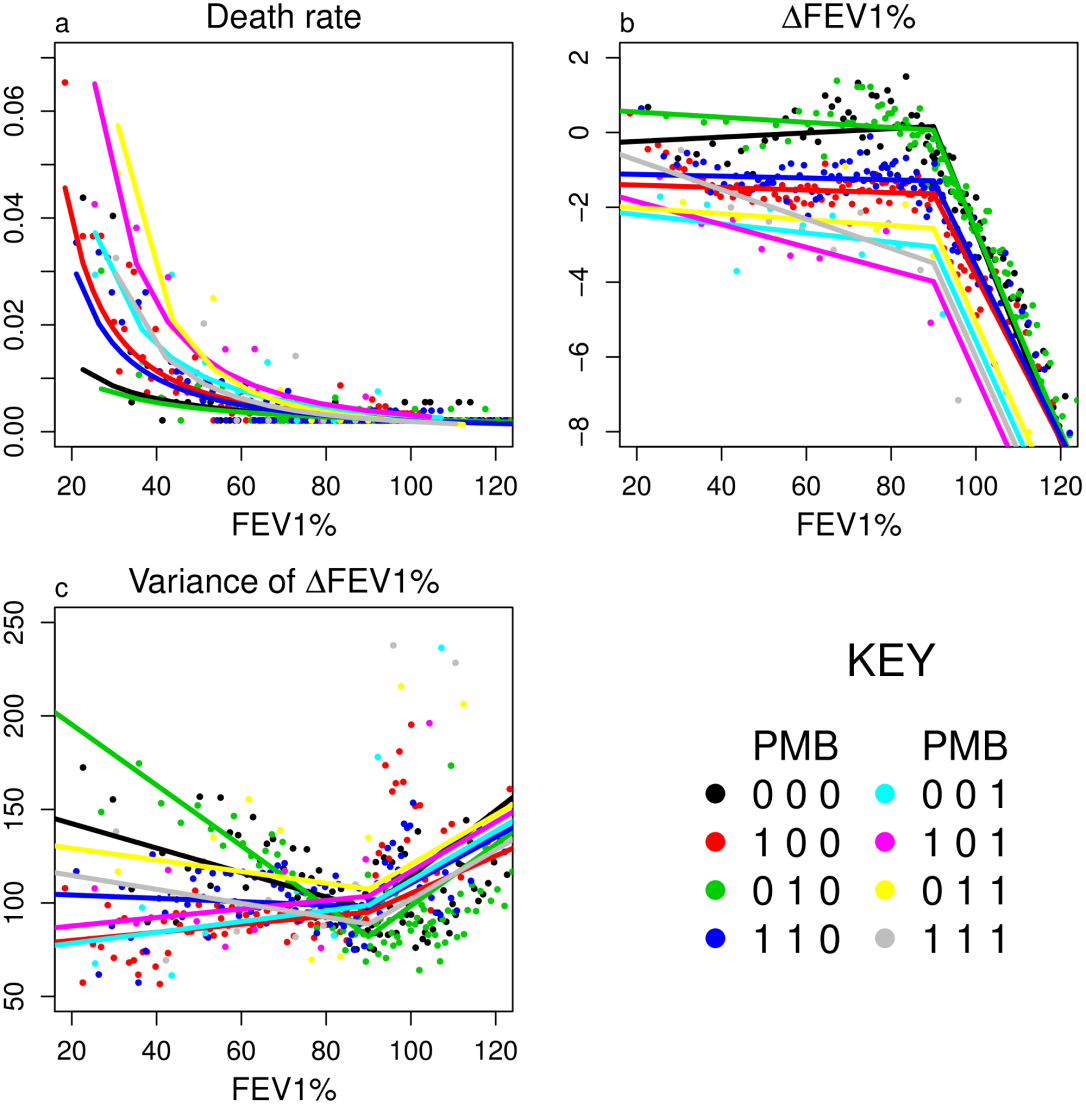
Annual death rate, annual change in FEV1% (ΔFEV1%), and variance of ΔFEV1% as a function of current FEV1% and bacterial infection state. Colors indicate the current bacterial status of patients based on testing for *P. aeruginosa*, MSSA and *Burkholderia* (given as PMB in the legend, with 1 and 0 indicating a positive or negative test respectively). Each dot shows the average value for patients broken into FEV1% bins by quantile for each bacterial status group, and the curves show the untransformed results of linear regression of log transformed mortality against log FEV1% (a), and piecewise linear fits with a breakpoint at FEV1% = 90% (b-c).

**Fig 3:**
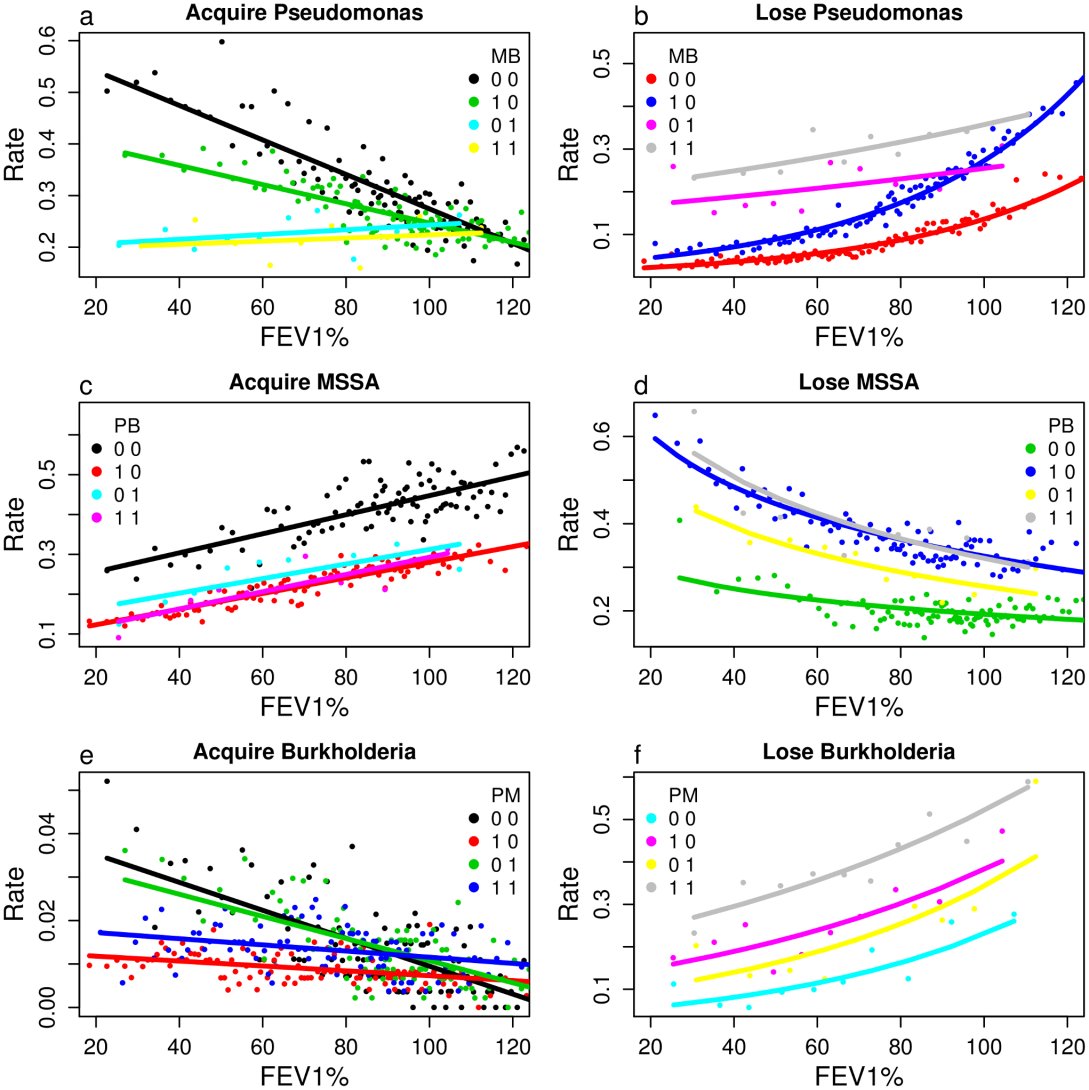
Annual rates of acquiring or losing *P. aeruginosa*, MSSA, and *Burkholderia* as a function of current FEV1% and bacterial infection state. Colors indicate current infection status, with each panel including only those consistent with the transition under consideration. As in Fig 2, each dot shows the measured rate in each set of patients binned into FEV1% groups, and curves fit data as described in the Methods.

Our equations track the density *u*_*s*_(*x*, *t*) of patients, where *x* represents the current FEV1%, *t* the time in years, and *s* the current bacterial carriage state. The distribution of FEV1% values in the subsequent year conditional on its value in the current year is well-approximated by a normal distribution (S1 Fig), justifying the use of a diffusion model rather than an integrodifferential equation [36]. The equations take the form

$$\begin{array}{ccc} \frac{\partial u_{s}}{\partial t} & = & \frac{\partial }{\partial x}D_{s}\left(x\right)\frac{\partial u_{s}}{\partial x}-\frac{\partial }{\partial x}v_{s}\left(x\right)u_{s}\\ & & \;\;\;\;\;\;-\delta_{s}\left(x\right)u_{s}-\sum_{s^{\prime }\neq s}\rho_{s\to s^{\prime }}u_{s}+\sum_{s^{\prime }\neq s}\rho_{s^{\prime }\to s}u_{s^{\prime }} \end{array}$$ (1)

where *s* and *s*′ represent bacterial carriage states. *D*_*s*_(*x*) is the diffusion coefficient (computed as 0.5 times the variance (Fig 2c)), *v*_*s*_(*x*) is the advection term found from the expected change in FEV1% (Fig 2b), *δ*_*s*_(*x*) is the death rate (Fig 2a), *ρ*_*s* → *s*′_(*x*) is the rate of transition from state *s* to state *s*′ due to pathogen acquisition or loss (Fig 3).

We numerically solve these equations with the ReacTrans package in R [35]. Initial conditions are normal distributions at ages less than 10 matched to the data in each of the eight possible bacterial carriage states (S2 Fig, S1 Table).

We compare survivorship with Kaplan-Meier curves (the survfit function in R) for both real patients and model output, and FEV1% and the fraction of patients infected visually using the supsmu function in R. As a more formal comparison, we compute the likelihood of the data given the model. Our likelihood incorporates all three model components: survivorship, FEV1%, and bacterial carriage state. From the model output, we find the probability that a patient has a particular age, bacterial carriage state, and FEV1% rounded to the nearest integer. We sum the natural logs of those probabilities over all patients alive in 2010 with data on microbiology and FEV1% to find the log likelihood associated with FEV1% and bacterial carriage. To avoid repeating patients, we find the log likelihood associated with survivorship from the start and end date for each patient. We sum the log probability of survivorship until 2011 or of death based on the survival curve computed from the model.

## Results

Restricting to pairs of consecutive years for a given patient with sufficient data to compute FEV1% measurements, complete microbiology, and no prior transplant, the Cystic Fibrosis Foundation Patient Registry (CFFPR) during the years 1996–2011 includes data on 207,363 annual transitions, representing 28,538 patients with 1 to 15 years of data for each (mean 7.27, summarized in Table 2). Without missing data, the total number of possible transitions for these patients during these years is 215,946, giving us 96.0% complete data. Including transplant patients increases the number of annual transitions with complete data to 213,497, or an increase of less than 3%.

**Table 2:** Summary of characteristics of patients used in the analysis.

From the 1996–2011 CFFPR					
age range	number of data points	FEV1%	*P. aeruginosa*	MSSA	*Burkholderia*
age ≤ 10	40331	101.8	0.389	0.572	0.0140
10 < age ≤ 18	79722	84.2	0.583	0.561	0.0289
18 < age ≤ 25	40991	70.7	0.765	0.449	0.0503
25 < age ≤ 35	28025	60.9	0.814	0.366	0.0579
35 < age	18294	56.5	0.782	0.312	0.0435
From the 1986–1995 CFFPR					
age ≤ 10	16228	87.9	0.536	0.338	0.0132
10 < age ≤ 18	28316	69.3	0.706	0.356	0.0396
18 < age ≤ 25	14650	55.3	0.801	0.291	0.0639
25 < age ≤ 35	10146	48.2	0.818	0.244	0.0505
35 < age	3133	44.8	0.794	0.219	0.0361

From the years 1986–1995, we have data on 72,473 annual transitions, representing 16,619 patients with 1 to 10 years of data for each (mean 4.36). The total number of possible transitions for these patients during these years is 79,915, giving us 90.7% complete data. Including transplant patients increases the number of annual transitions with complete data to 73,510, an increase of 1.4%.

FEV1% shows strong and consistent relationships with one-year survivorship, annual changes in FEV1%, and bacterial carriage status (Figs 2 and 3 and S1 Table). Low FEV1% is strongly associated with mortality, particularly in combination with *Burkholderia* (Fig 2a). Patients with FEV1% exceeding 90% show a large average decrease, which may be partially attributable to measurement errors, however the finding is consistent with observations among patients with normal or high lung function [25]. Variance in future FEV1% is always large, with a standard deviation of roughly 10% that dominates the typically small average change (Fig 2c). Patients with no bacteria or with only MSSA show little if any average loss of FEV1%, while those with *P. aeruginosa* lose an average of approximately 2% per year, and those with *Burkholderia* even more (Fig 2b). Low FEV1% predicts increased acquisition and decreased loss of *P. aeruginosa* (Fig 3a and 3b) and *Burkholderia* (Fig 3c and 3d), and decreased acquisition and increased loss of MSSA (Fig 3e and 3f).

Interactions between bacteria appear as differences in the elevations of the lines. The highest rates of acquiring and lowest rates of losing *P. aeruginosa* occur for patients without *Burkholderia* Fig 3a and 3b). Similarly, the highest rates of acquiring and lowest rates of losing MSSA occur in patients without *P. aeruginosa* Fig 3c and 3d), and the highest rate of acquiring *Burkholderia* in patients without *P. aeruginosa* Fig 3e).

**Fig 4:**
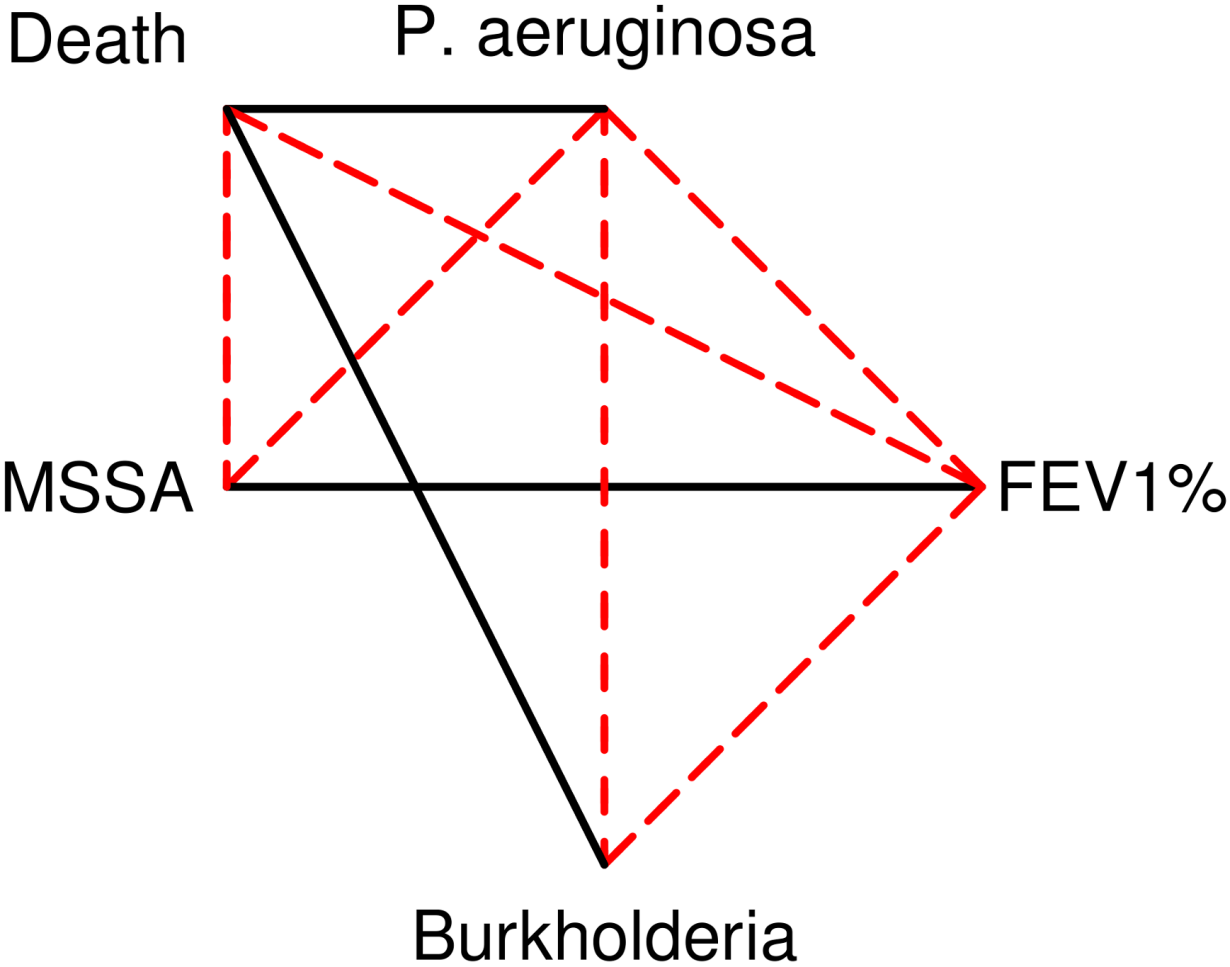
Significant interactions among state variables. Positive interactions are indicated by solid black lines and negative interactions by dashed red lines. Except for mortality, all relationships are reciprocal. For example, carriage of *P. aeruginosa* leads to reduction of FEV1%, and higher values of FEV1% lead to reduced carriage of *P. aeruginosa*.

These interactions consist of one positive feedback loop between two damaging factors (*P. aeruginosa* with low FEV1%), two negative feedback loops between bacteria (*P. aeruginosa* with MSSA and *P. aeruginosa* with *Burkholderia*), and one positive feedback loop between two protective factors (MSSA and high FEV1%) (Fig 4).

**Fig 5:**
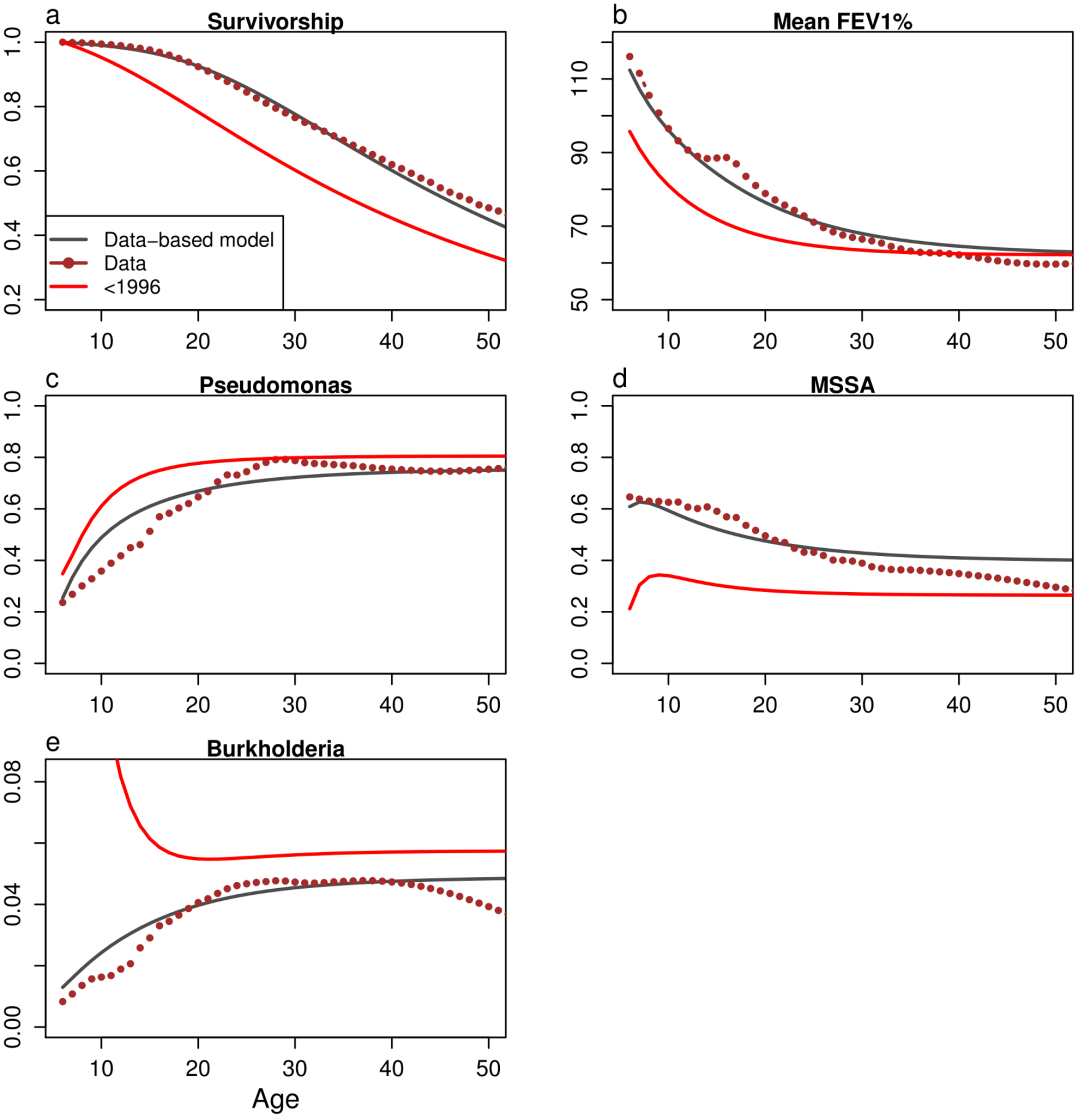
Predicted and observed survivorship, progression of FEV1%, and prevalence of the three main infections as functions of patient age. The dots indicate the values in data from 2010, and the red line is the model based on data from patients before 1996.

The numerical solution of Eq 1 accurately predicts the distribution of FEV1% for patients of a particular age and bacterial carriage state (hypothesis H1, S4 Fig). If the models are based on the average rather than the maximum annual FEV%, model results also closely track the data (results not shown). These distributions can be summarized as averages (Fig 5). Using parameter values from 1986–1995 predicts poorer survivorship, higher levels of *P. aeruginosa* and lower MSSA, consistent with data [37, 38], and indicating the lifelong effects of improvements in treatment.

**Fig 6:**
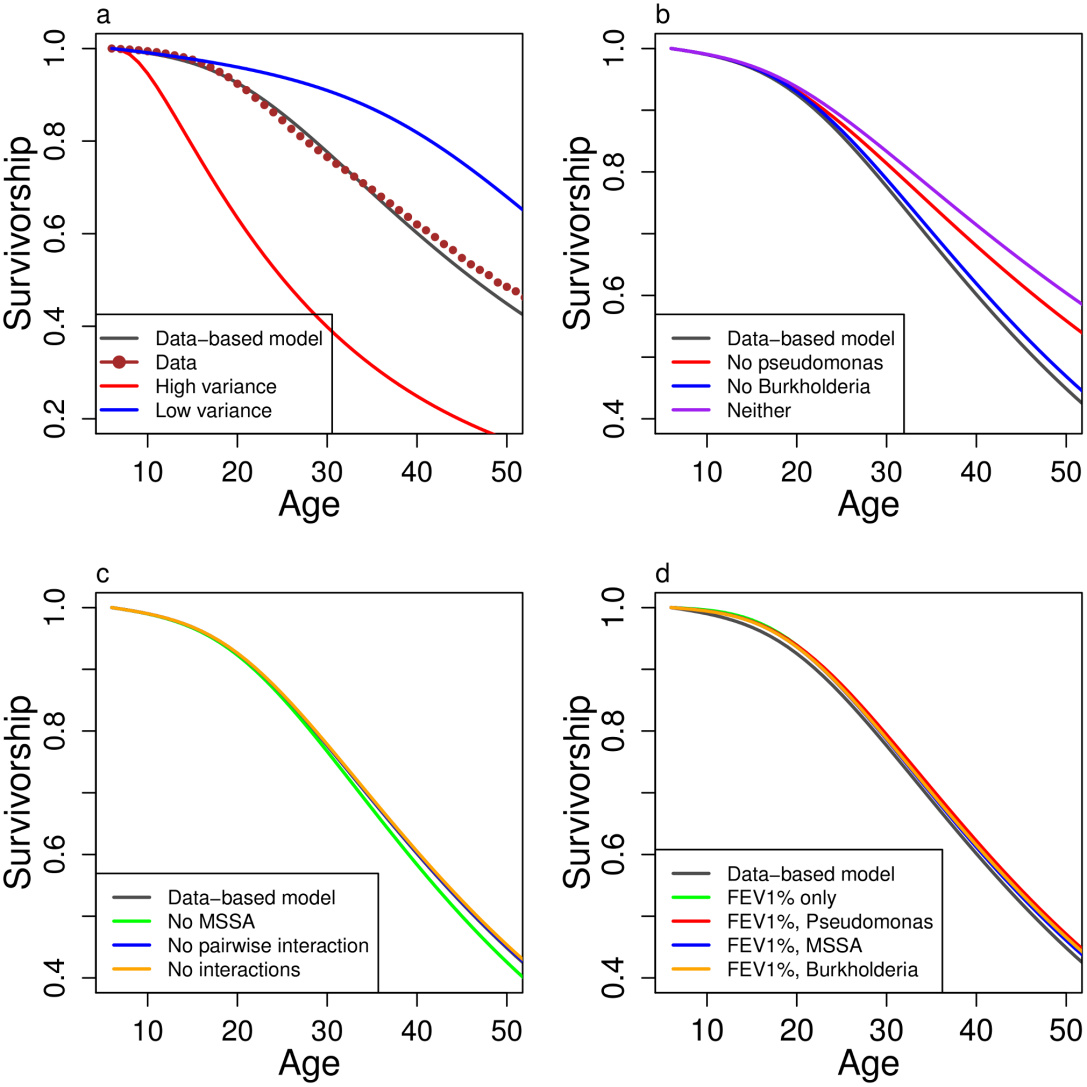
Survivorship comparing different model assumptions and strategies. We compare the data-based model with models that **a.** increase variance by a factor of 5 (High variance) or decrease variance by a factor of 5 (Low variance), **b.** eliminate *P. aeruginosa*, eliminate *Burkholderia*, or eliminate both *P. aeruginosa* and *Burkholderia*, **c.** eliminate MSSA, eliminate the interaction of MSSA with *P. aeruginosa* (No pairwise interaction), or eliminate all bacterial interactions (No interactions), **d.** include only the given subset of variables.

We use the model to test hypotheses H2–H4 presented in the introduction. First (H2), the observed high annual variance in the change in FEV1% contributes strongly to the poor survivorship of the population (Fig 6a). This results from the non-linearity in the mortality curve, with high variance being more likely to dip patients, even if transiently, into the danger zone of low FEV1% where death is more likely (S5 Fig). Second (H3), elimination of *P. aeruginosa* predicts a significant increase in survivorship, and the additional elimination of *Burkholderia* provides a synergistic benefit (Fig 6b). This synergism results from the predicted increase in *Burkholderia* when released from competition with *P. aeruginosa* (S6 Fig). Elimination of MSSA leads to only a small predicted improvement in survival. Finally, and contrary to our original hypothesis (H4), removal of bacterial interactions produces essentially no change in predicted survival (Fig 6c), as does removal of only the interaction between MSSA and *P. aeruginosa*. This indicates that the main benefits of MSSA might be direct, through its reduction of loss of FEV1% and mortality, rather than indirect through competition with *P. aeruginosa* (S7 Fig).

We also compare survivorship with four lower dimensional models: using only FEV1%, FEV1% and *P. aeruginosa*, FEV1% and MSSA, and FEV1% and *Burkholderia* (parameter values in S3 Table). These models predict survivorship (Fig 6d) and FEV1% well (S8 Fig).

**Fig 7:**
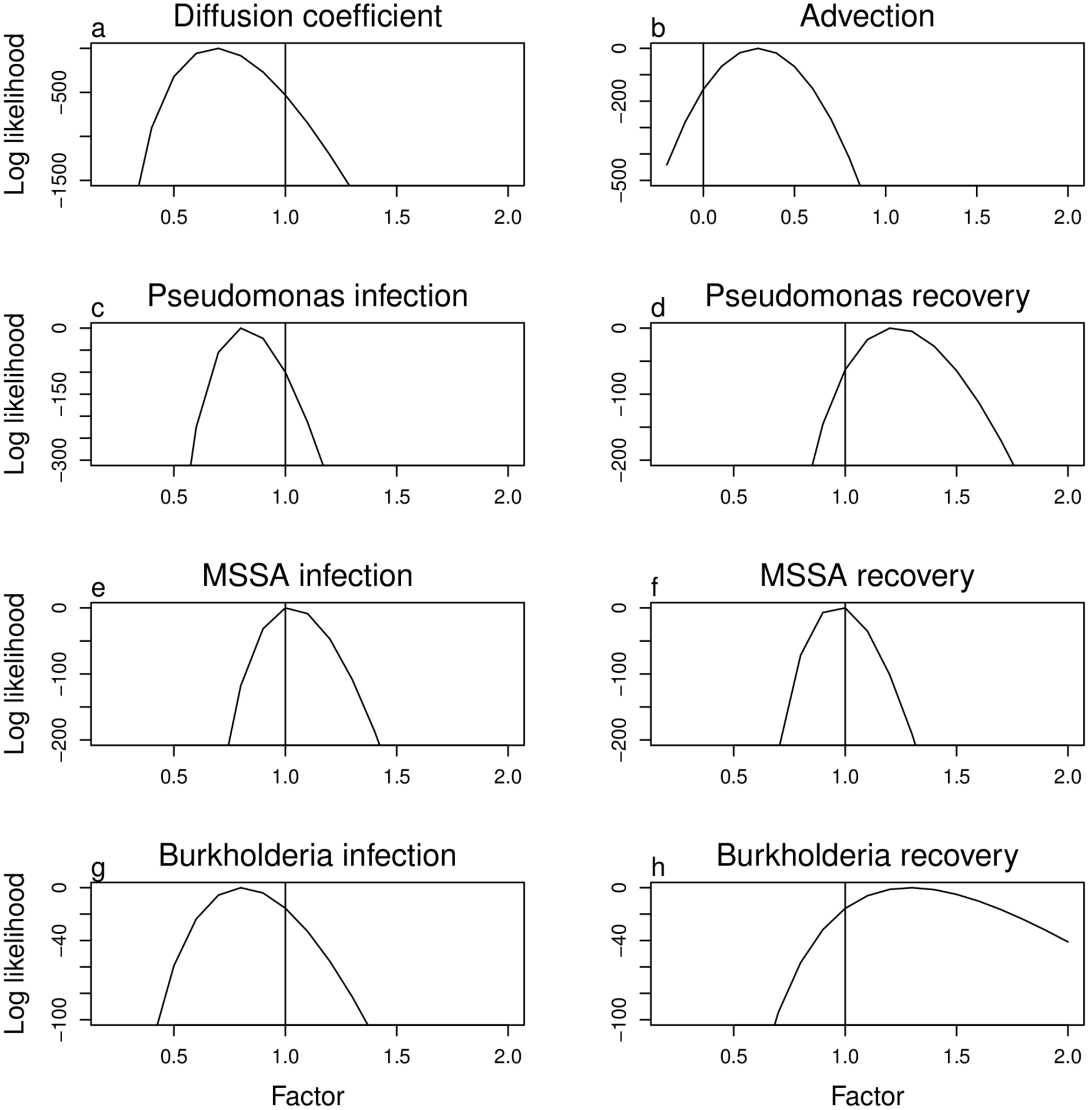
Log likelihood of the data as a function of altered parameter values. The horizontal axis shows change in the basic value of the given parameter by a multiplicative factor, except for the advection coefficient in **b** which is altered by adding the given amount. The vertical axis is the difference in the log likelihood from the maximum for that parameter. The likelihood associated with the FEV1% is reduced in part by the wiggle created in FEV1% when the formula for transformation to percent predicted switches from pediatric to adult [39] (Fig 5b).

We use the likelihood to test whether our parameter values, estimated directly from the data, provide the best fit to the data. Although the baseline parameters are rarely the best, they are consistently close (Fig 7). In particular, models with lower diffusion and slower loss of FEV1% provide better fits to the full course of data, likely due to lack of explicit inclusion of measurement error in the models (see Discussion).

## Discussion

Starting with summaries of annual changes in pulmonary function, bacterial acquisition, and bacterial loss from data in the Cystic Fibrosis Foundation Patient Registry (CFFPR), we develop a system of partial differential equations to predict the course of lung function, bacterial infection and survival in the population of patients with cystic fibrosis in the United States. The equations follow the distribution of a key measure of lung function, FEV1%, and carriage of any possible subset of three key bacteria, *P. aeruginosa*, methicillin-sensitive *S. aureus* (MSSA), and members of the *Burkholderia* complex. We find that FEV1% is highly variable, with the standard deviation of annual change greatly exceeding the mean annual change. The data show that carriage of the damaging bacteria *P. aeruginosa* and *Burkholderia* complex contribute to more rapid decrease in FEV1% and higher mortality. In turn, lower FEV1% enhances acquisition of these bacteria, reduces their rate of loss, and increases mortality. MSSA, on the other hand, reduces annual FEV1% decline and the rate of mortality, while being acquired most quickly and lost most slowly at high FEV1%.

The data suggest that *P. aeruginosa* and MSSA have a strong antagonistic interaction, with each reducing the rate of acquisition and increasing the rate of loss of the other. *Burkholderia* complex shows a similar antagonism with *P. aeruginosa* but no significant interaction with MSSA. We summarize these connections as one damaging positive feedback loop (*P. aeruginosa* with low FEV1%), two negative feedback loops (*P. aeruginosa* with MSSA and with *Burkholderia*), and one protective positive feedback loop (MSSA with high FEV1%) (Fig 4).

Models incorporating these interactions into a system of partial differential equations match the long-term behavior of the population closely (Fig 5). The models support the hypothesis that the observed high variance in FEV1% contributes to the overall poor survivorship in the population (Fig 6a) and that existing models of survival in CF [8, 13, 17] may be improved by including some measure of variance. Eliminating infection by both *P. aeruginosa* and *Burkholderia* predicts improved survivorship and FEV1%, while elimination of MSSA reduces survivorship. Contrary to our original hypothesis, removing the interaction between *P. aeruginosa* and MSSA from the model predicts almost no change in the course of disease (Fig 6c).

Statistical and medical studies have identified many other covariates, such as severity of the CF mutation [40], pancreatic sufficiency, CF related diabetes, weight, number of acute exacerbations, and other infections, particularly methicillin-resistant *S. aureus*, or MRSA, that affect changes in FEV1% and survivorship [8, 17, 24]. Although FEV1% is a powerful predictor, alternative measures of lung function might provide additional information about disease progression [24]. Emerging pathogens could further complicate the picture by increasing the dimensionality of the model and the number of potential interactions [41]. We do not include the considerable diversity within *P. aeruginosa* [42–44], nor the progression from non-mucoid to mucoid types that is associated with poor outcomes [18, 26, 45]. Recent evidence indicates that some members of the *Burkholderia* complex can also diversify in long-term CF patients [46, 47]. Where sufficient data are available, we are working to extend the models to include these factors, including the effects of interventions.

A recent paper uses threshold regression techniques to develop a one-year survival model in cystic fibrosis [48], with the central innovation being to impose stochastic shocks onto a declining CF health index, with mortality occurring when shocks drop the health index below a critical threshold. The model presented here combines stochastic decay to implicitly model shocks through the highly non-linear probability of death at low FEV1%. We are working to develop a framework to unify these methods.

Our models treat the population of CF patients as a homogeneous cohort conditional on the covariates included [24]. Our models absorb other components of this heterogeneity by looking at the dynamics of the population. We treat FEV1% as a sufficient surrogate biomarker to characterize the stage of the disease without explicitly including age [49], thus assuming that the future course is independent of the past. Models including age explicitly do show a better fit to to the progression of MSSA, but with little effect on the overall dynamics (results not shown). We exclude transplanted patients who have a quite different course of disease and associated causes of death, but aim to extend the models to include transition to the transplanted population and the resulting dynamics within that additional class of patients.

This model does not explicitly include measurement error in FEV1%, which falls in the range of 4.5–6.3 FEV1% units [23, 50]. Using multiple measurements per year allows us to estimate the error at 5.8%, within this range. Models which correct for this in our estimates of the variance in the change in FEV1% fit the data well, but require additional correction factors in the functions for mortality and and bacterial carriage. We are currently developing the methods to extend the modeling approach to fully incorporate measurement error in FEV1% and false positives and false negatives in the microbiology. As an additional check, we repeated the analyses using the mean rather than maximum FEV1%, based on a median of three measurements per year, and found that the conclusions of the original analysis hold up, although the magnitude of the observed variability in FEV1% is lower.

These models neglect multiyear associations among measurements [23], particularly for bacterial infections where a positive test is much more likely after two consecutive years with positive tests than after only one. Study of MRSA indicates that patients with persistent MRSA show stronger declines of FEV1% than those with transient infections [51] and greater reduction in survival [52]. Our current methods do not distinguish between transient and established infections, a close parallel with the clinical situation, and we are developing extensions to correct for measurement errors in order to be able to clearly define these alternative states.

These models provide a transparent way to summarize data and identify important trends in disease dynamics, and an efficient modeling framework for predicting the population-level consequences of potential medical interventions where experiments are impossible. Our key findings about the importance of variance and the beneficial effects of MSSA point the way toward a more mechanistic understanding of this complex disease.

## Supporting Information

S1 Fig Smoothed density of FEV1% in the next year. The panels show patients with current FEV1% within 0.5 of **a.** 30%, **b.** 50%, **c.** 70%, and **d.** 100%. The dashed vertical red line is the average of FEV1% in the current year, and the solid vertical green line the average of this patient cohort in the next year.
(TIFF)

S2 Fig Distribution of FEV1% for patients entering the database with age less than 10 for the 8 possible infection states. The states are designated in the titles as in the legend to Fig 3.
(TIFF)

S3 Fig Input data for lower dimensional models. The first column shows mortality, variance in ΔFEV1% and ΔFEV1% for a model that tracks only FEV1%. The next columns present models that track FEV1% and *P. aeruginosa*, MSSA, and *Burkholderia* respectively, including a panel with acquisition (colored points and curve) or loss (black points and curve) of infection.
(TIFF)

S4 Fig Distribution of predicted (solid lines) and observed (dashed lines) FEV1% at four ages for all patients pooled and for those testing positive for each of the three infections. Distributions are normalized to integrate to the total number of patients in that category.
(TIFF)

S5 Fig Trajectories predicted by the model with altered variance. Variance is increased by a factor of 5 (High variance) or decreased by a factor of 5 (Low variance) of **a.** FEV1%, **b.** *P. aeruginosa*, **c.** MSSA, and **d.** *Burkholderia*.
(TIFF)

S6 Fig Trajectories predicted by the model with *P. aeruginosa* acquisition eliminated, *Burkholderia* acquisition eliminated, or when both have been eliminated. (TIFF)

S7 Fig Trajectories predicted by the model with MSSA acquisition eliminated, without the pairwise interaction of MSSA and *P. aeruginosa*, and without any bacterial interactions. (TIFF)

S8 Fig Trajectories predicted by lower dimensional models with just FEV1%, FEV1% and *P. aeruginosa*, FEV1% and MSSA, and FEV1% and *Burkholderia*. (TIFF)

S1 Table Coefficients of the full model. (PDF)

S2 Table Coefficients of the full model with patients from 1986–1995. (PDF)

S3 Table Coefficients of the lower dimensional models. (PDF)
