# Supplementary figures and images for: The Dynamics of Disease Progression in Cystic Fibrosis
Source: PLoS One. 2016 Jun 1;11(6):e0156752. doi: 10.1371/journal.pone.0156752 (PMC4889102; doi:10.1371/journal.pone.0156752)

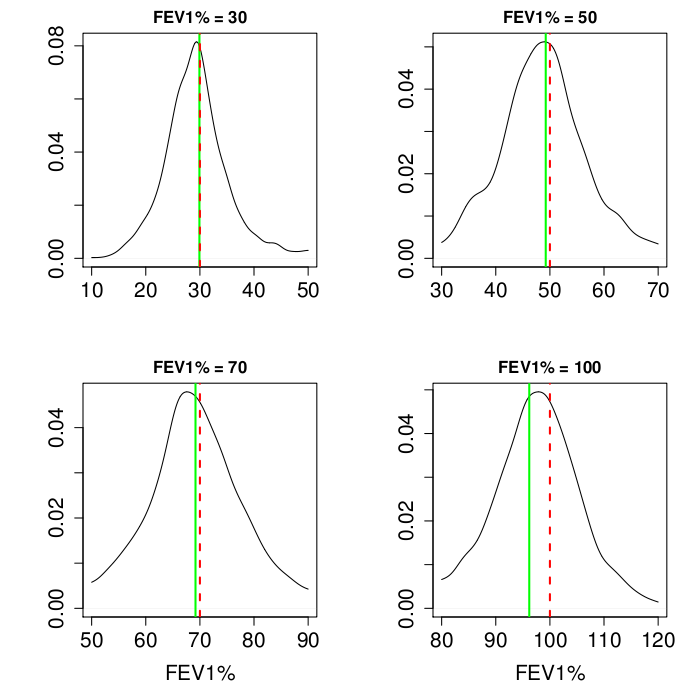

Supplement: S1 Fig — The panels show patients with current FEV1% within 0.5 of a. 30%, b. 50%, c. 70%, and d. 100%. The dashed vertical red line is the average of FEV1% in the current year, and the solid vertical green line the average of this patient cohort in the next year. (TIFF) [file pone.0156752.s001.tiff]

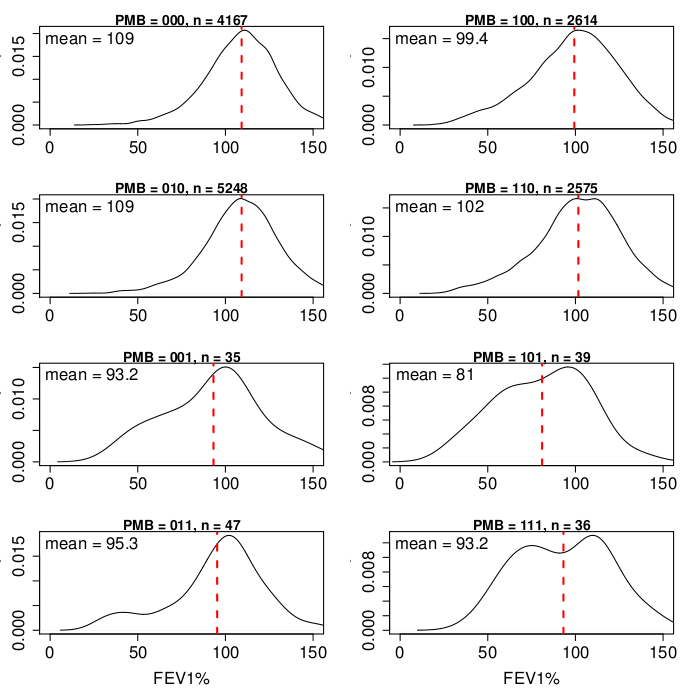

Supplement: S2 Fig — The states are designated in the titles as in the legend to Fig 3. (TIFF) [file pone.0156752.s002.tiff]

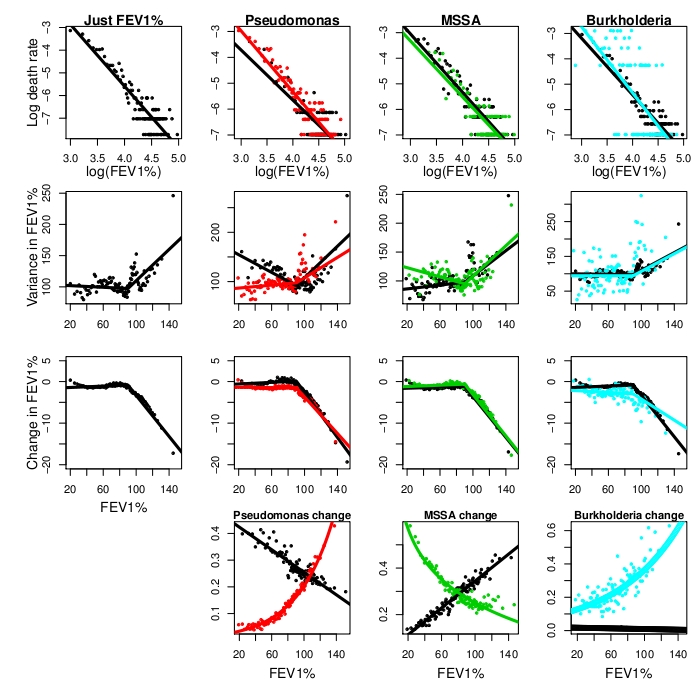

Supplement: S3 Fig — The first column shows mortality, variance in ΔFEV1% and ΔFEV1% for a model that tracks only FEV1%. The next columns present models that track FEV1% and P. aeruginosa, MSSA, and Burkholderia respectively, including a panel with acquisition (colored points and curve) or loss (black points and curve) of infection. (TIFF) [file pone.0156752.s003.tiff]

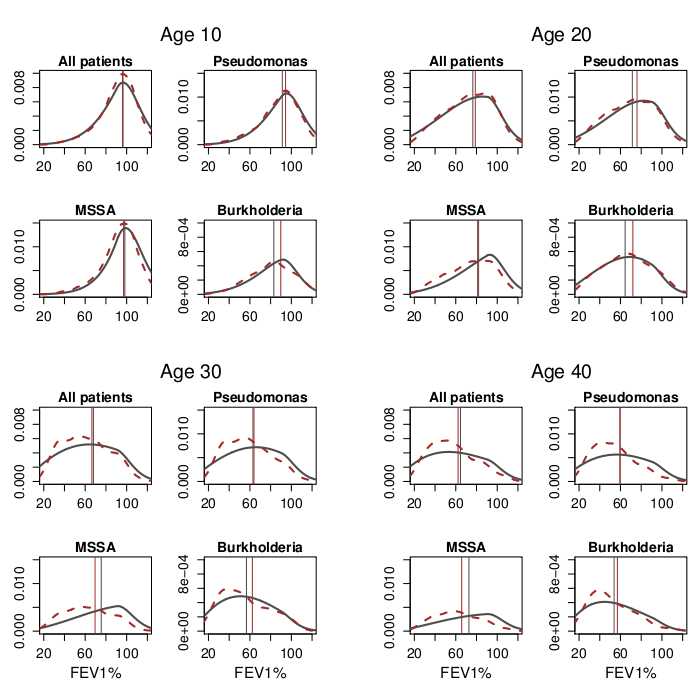

Supplement: S4 Fig — Distributions are normalized to integrate to the total number of patients in that category. (TIFF) [file pone.0156752.s004.tiff]

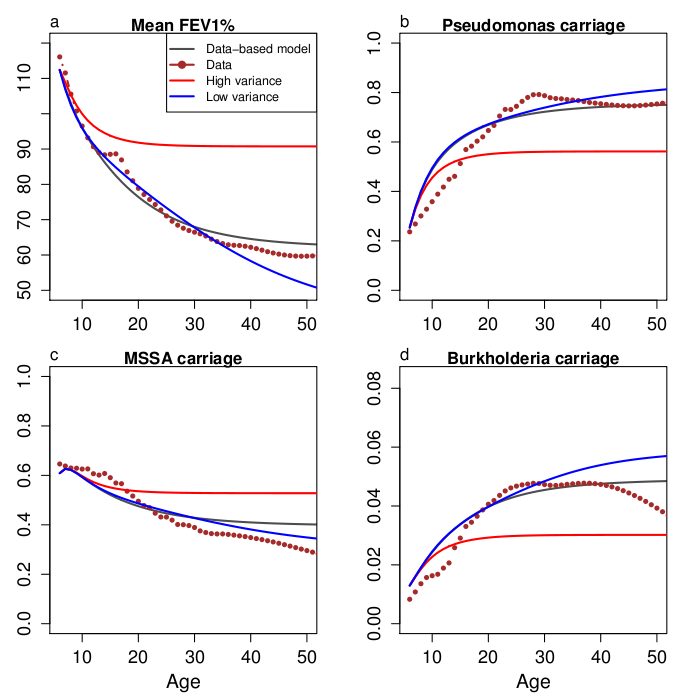

Supplement: S5 Fig — Variance is increased by a factor of 5 (High variance) or decreased by a factor of 5 (Low variance) of a. FEV1%, b. P. aeruginosa, c. MSSA, and d. Burkholderia. (TIFF) [file pone.0156752.s005.tiff]

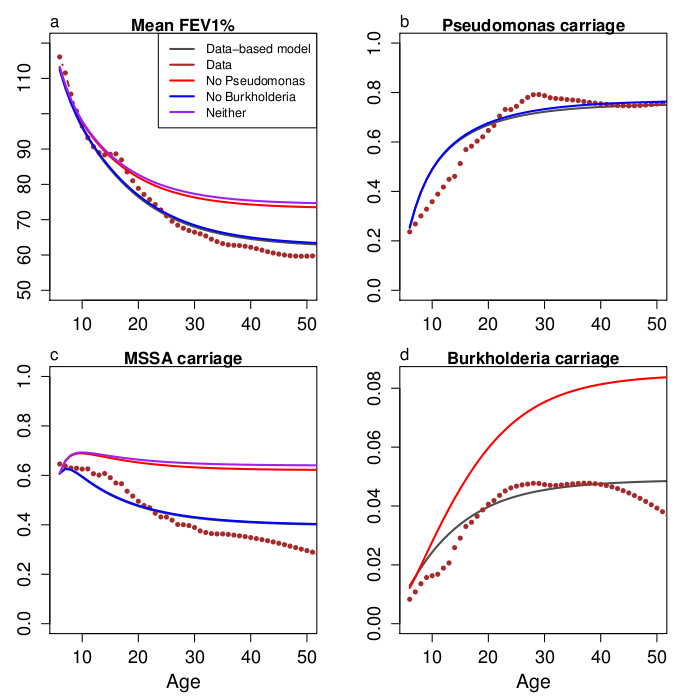

Supplement: S6 Fig — (TIFF) [file pone.0156752.s006.tiff]

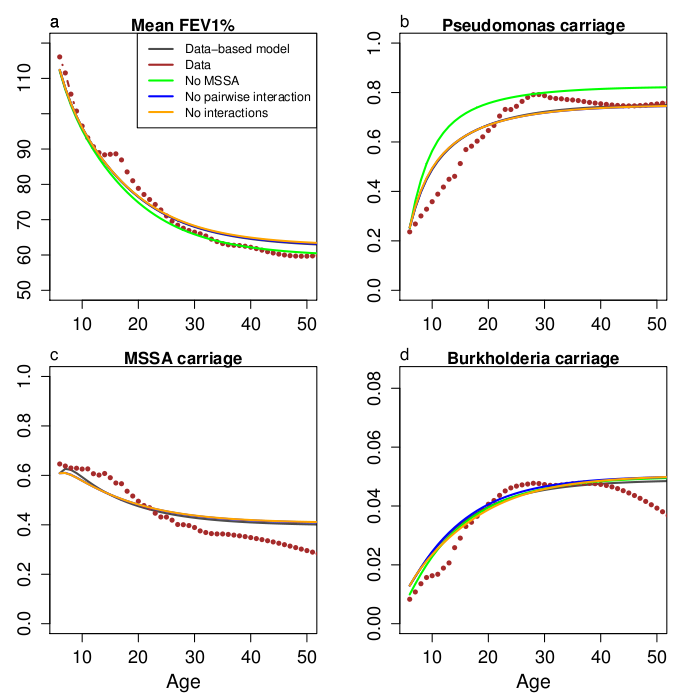

Supplement: S7 Fig — (TIFF) [file pone.0156752.s007.tiff]

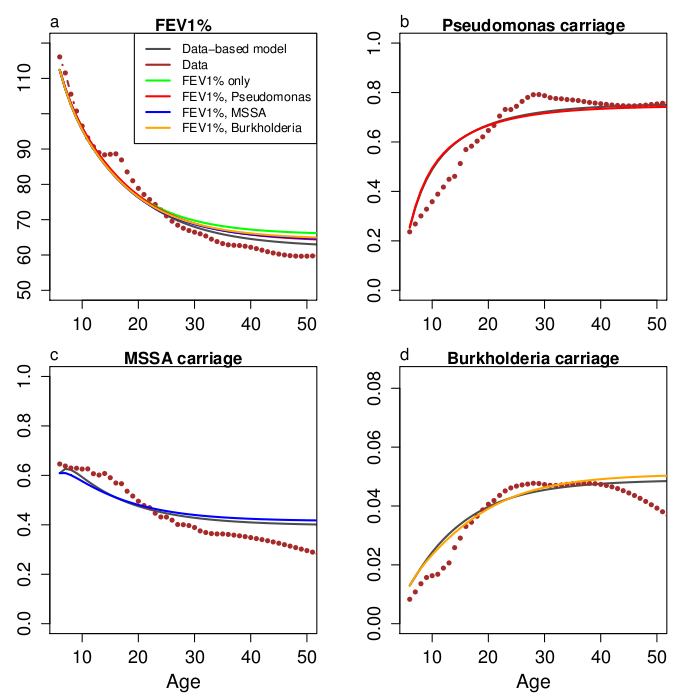

Supplement: S8 Fig — (TIFF) [file pone.0156752.s008.tiff]
