# Supplementary material for: The Dynamics of Disease Progression in Cystic Fibrosis
Source: PLoS One. 2016 Jun 1;11(6):e0156752. doi: 10.1371/journal.pone.0156752 (PMC4889102; doi:10.1371/journal.pone.0156752)
Supplement: S1 Table — (PDF) [file pone.0156752.s009.pdf]

Table S1. Coefficients of the full model. The state describes whether (1) or not (0) a patient is infected with *P. aeruginosa*, MSSA, or *Burkholderia* in that order. The subscripts I, s and $s2$ represent the intercept, slope, and slope with FEV1% > 90 (when relevant), respectively. μ stands for mortality rate, ΔFEV1% for mean change in FEV1%, *Pseudo* for the rate of change in *P. aeruginosa* state, MSSA for the rate of change in MSSA state, *Burk* for the rate of change in *Burkholderia* state, and Var(FEV1%) for the variance in next year's FEV1%. The final columns give the mean, standard deviation of FEV1% in young patients who are entering the data base, and the fraction of patients in each category.

state	Patients	μ_I	μ_s	$\Delta FEV1\%_I$	$\Delta FEV1\%_s$	$\Delta FEV1\%_{s2}$
000	29331	-0.99	-1.109	-0.352	0.00568	-0.281
100	76670	2.118	-1.787	-1.338	-0.00336	-0.219
010	47085	-1.615	-0.975	0.683	-0.00686	-0.268
110	54914	1.635	-1.692	-1.070	-0.00246	-0.228
001	1855	2.705	-1.851	-1.949	-0.0123	-0.252
101	2265	4.453	-2.221	-1.235	-0.0306	-0.252
011	1769	7.053	-2.889	-1.857	-0.00783	-0.252
111	1670	4.361	-2.308	0.041	-0.0392	-0.252
state	$Pseudo_I$	$Pseudo_s$	$MSSA_I$	$MSSA_s$	$Burk_I$	$Burk_s$
000	0.608	-0.00334	0.2080	0.00239	0.0416	-3.21e-04
100	-4.218	0.0222	0.0840	0.00196	0.0129	-5.62e-05
010	0.433	-0.00186	0.4860	-0.06370	0.0362	-2.55e-04
110	-3.539	0.0224	1.1200	-0.17300	0.0187	-7.10e-05
001	0.197	0.000459	0.1290	0.00183	-3.2100	1.74e-02
101	-1.871	0.00503	0.0765	0.00216	-2.1300	1.17e-02
011	0.194	0.000303	0.9340	-0.14700	-2.5700	1.50e-02
111	-1.632	0.00601	1.2500	-0.20300	-1.6000	9.48e-03
state	$Var(FEV1\%)_I$	$Var(FEV1\%)_s$	$Var(FEV1\%)_{s2}$	$\overline{FEV1\%}$	sd FEV1%	fraction
000	155.5	-0.653	1.756	112.70	21.50	0.29020
100	75.65	0.216	1.002	111.20	23.45	0.09715
010	227.8	-1.620	1.645	113.70	19.47	0.44690
110	106.02	-0.0885	1.240	110.70	21.76	0.15280
001	72.69	0.289	1.324	91.91	23.33	0.00259
101	83.12	0.228	1.324	91.91	23.33	0.00129
011	135.46	-0.311	1.324	91.91	23.33	0.00648
111	122.11	-0.371	1.324	91.91	23.33	0.00259
