# Supplementary material for: The Dynamics of Disease Progression in Cystic Fibrosis
Source: PLoS One. 2016 Jun 1;11(6):e0156752. doi: 10.1371/journal.pone.0156752 (PMC4889102; doi:10.1371/journal.pone.0156752)
Supplement: S2 Table — (PDF) [file pone.0156752.s010.pdf]

Table S2. Coefficients of the full model with patients from 1986-1995, notation as in Table S1.

state	Patients	μ_I	μ_s	ΔFEV1%$_I$	ΔFEV1%$_s$	ΔFEV1%$_{s2}$
000	11686	-2.59	-0.478	6.18	-0.0694	-0.33
100	39754	-1.27	-0.776	3.28	-0.077	-0.244
010	10547	-3.45	-0.286	8.75	-0.111	-0.281
110	13435	-1.63	-0.704	4.05	-0.0832	-0.268
001	1092	0.632	-1.16	1.46	-0.0664	-0.274
101	1112	2.57	-1.7	1.51	-0.0809	-0.274
011	375	1.31	-1.44	7.07	-0.168	-0.274
111	454	-2.5	-0.449	2.63	-0.111	-0.274
state	$Pseudo_I$	$Pseudo_s$	MSSA$_I$	MSSA$_s$	$Burk_I$	$Burk_s$
000	1.03	-0.00714	0.22	0.00146	0.0638	-4.69e-4
100	-3.51	0.0146	0.0722	0.00151	0.0261	1.36e-4
010	0.621	-0.00364	0.456	-0.0221	0.0621	-4.85e-4
110	-2.77	0.0106	0.864	-0.0729	0.0337	-1.48e-4
001	0.413	-0.00111	0.113	0.000792	-1.93	7.09e-3
101	-1.57	0.00673	0.108	0.00116	-1.62	1.09e-2
011	0.27	0.000595	1.19	-0.162	-2.35	1.96e-2
111	-1.23	-0.000179	1.58	-0.227	-1.03	5.79e-3
state	Var(FEV1%)$_I$	Var(FEV1%)$_s$	Var(FEV1%)$_{s2}$	$\overline{\text{FEV1\%}}$	sd FEV1%	fraction
000	332	-1.89	2.54	104	24.7	0.353
100	122	0.864	1.18	86.8	26.8	0.338
010	408	-3.03	2.45	100	24.8	0.208
110	199	-0.387	1.74	87.8	28.8	0.0906
001	119	0.752	1.59	71.4	26.7	0.00388
101	55.2	1.69	1.59	71.4	26.7	0.00647
011	123	0.869	1.59	71.4	26.7	0.0
111	167	0.195	1.59	71.4	26.7	0.0
