# Supplementary material for: The Dynamics of Disease Progression in Cystic Fibrosis
Source: PLoS One. 2016 Jun 1;11(6):e0156752. doi: 10.1371/journal.pone.0156752 (PMC4889102; doi:10.1371/journal.pone.0156752)
Supplement: S3 Table — (PDF) [file pone.0156752.s011.pdf]

Table S3. Coefficients of the lower dimensional models, using the notation from Table S1. The first column gives results with a model using only FEV1%, the next two columns the rates for a model using FEV1% and *P. aeruginosa* for patients with and without infection, the next two with FEV1% and MSSA, and the last with FEV1% with and without *Burkholderia*.

coefficient	FEV1%	*Pseudo*=0	*Pseudo*=1	MSSA=0	MSSA=1	*Burk*=0	*Burk*=1
Patients	225347	80040	135519	110121	105438	208000	7559
μ_I	5.303	2.088	4.074	3.812	3.250	3.488	5.060
μ_s	-2.722	-1.935	-2.351	-2.286	-2.190	-2.2279	-2.600
$\Delta\text{FEV1\%}_I$	-1.587	-0.779	-1.329	-1.735	-1.360	-1.575	-1.957
$\Delta\text{FEV1\%}_s$	0.008177	0.009725	-0.002107	0.006459	0.008106	0.008387	-0.01094
$\Delta\text{FEV1\%}_{s2}$	-0.2468	-0.2709	-0.2194	-0.2453	-0.2491	-0.2490	-0.1267
$\text{Var(FEV1\%)}_I$	103.3	172.6	83.78	82.89	130.97	100.39	94.20
$\text{Var(FEV1\%)}_s$	-0.06646	-0.8988	0.1513	0.1651	-0.400	-0.03374	0.0007516
$\text{Var(FEV1\%)}_{s2}$	1.252	1.603	1.0341	1.0938	1.3194	1.2685	1.2685
rate_I		0.4657	-3.828	0.05575	1.1610	0.01924	-2.322
rate_s		-0.00211	0.02234	0.002886	-0.1977	-9.859e-05	1.313e-02
$\overline{\text{FEV1\%}}$	112.41	113.07	110.50	112.07	112.64	112.68	91.91
sd FEV1%	20.99	20.39	22.59	22.10	20.26	20.84	23.33
fraction	1	0.7461	0.2539	0.3912	0.6088	0.9870	0.01295
